# Discontinuation and dose adjustment of metoprolol after metoprolol‐paroxetine/fluoxetine co‐prescription in Dutch elderly

**DOI:** 10.1002/pds.4422

**Published:** 2018-03-24

**Authors:** Muh. Akbar Bahar, Yuanyuan Wang, Jens H.J. Bos, Bob Wilffert, Eelko Hak

**Affiliations:** ^1^ Groningen Research Institute of Pharmacy, Department of PharmacoTherapy, ‐Epidemiology and ‐Economics University of Groningen Groningen The Netherlands; ^2^ Faculty of Pharmacy Hasanuddin University Makassar Indonesia; ^3^ Department of Clinical Pharmacy and Pharmacology University Medical Center Groningen Groningen The Netherlands

**Keywords:** citalopram, CYP2D6, drug‐drug interactions (DDI), metoprolol, mirtazapine, paroxetine/fluoxetine, pharmacoepidemiology

## Abstract

**Purpose:**

Co‐prescription of paroxetine/fluoxetine (a strong CYP2D6 inhibitor) in metoprolol (a CYP2D6 substrate) users is common, but data on the clinical consequences of this drug‐drug interaction are limited and inconclusive. Therefore, we assessed the effect of paroxetine/fluoxetine initiation on the existing treatment with metoprolol on the discontinuation and dose adjustment of metoprolol among elderly.

**Methods:**

We performed a cohort study using the University of Groningen IADB.nl prescription database (www.IADB.nl). We selected all elderly (≥60 years) who had ever been prescribed metoprolol and had a first co‐prescription of paroxetine/fluoxetine, citalopram (weak CYP2D6 inhibitor), or mirtazapine (negative control) from 1994 to 2015. The exposure group was metoprolol and paroxetine/fluoxetine co‐prescription, and the other groups acted as controls. The outcomes were early discontinuation and dose adjustment of metoprolol. Logistic regression was applied to estimate adjusted odds ratios (OR) and 95% confidence intervals (CI).

**Results:**

Combinations of metoprolol‐paroxetine/fluoxetine, metoprolol‐citalopram, and metoprolol‐mirtazapine were started in 528, 673, and 625 patients, respectively. Compared with metoprolol‐citalopram, metoprolol‐paroxetine/fluoxetine was not significantly associated with the early discontinuation and dose adjustment of metoprolol (OR = 1.07, 95% CI:0.77‐1.48; OR = 0.87, 95% CI:0.57‐1.33, respectively). In comparison with metoprolol‐mirtazapine, metoprolol‐paroxetine/fluoxetine was associated with a significant 43% relative increase in early discontinuation of metoprolol (OR = 1.43, 95% CI:1.01‐2.02) but no difference in the risk of dose adjustment. Stratified analysis by gender showed that women have a significantly high risk of metoprolol early discontinuation (OR = 1.62, 95% CI:1.03‐2.53).

**Conclusion:**

Paroxetine/fluoxetine initiation in metoprolol prescriptions, especially for female older patients, is associated with the risk of early discontinuation of metoprolol.

## 1 INTRODUCTION

Clinically relevant cytochrome P450 mediated drug‐drug interactions (DDI) are prevalent in geriatric patients with multiple comorbidities such as cardiovascular and psychiatric diseases.^1^, ^2^, ^3^, ^4^ Metoprolol and paroxetine/fluoxetine as the drugs of choice for treating these chronic illnesses consecutively are often observed to be co‐prescribed in the elderly.^5^, ^6^, ^7^ Several studies have reported that the combination triggers cytochrome P450 2D6 (CYP2D6) mediated pharmacokinetic DDI.^8^, ^9^, ^10^ Metoprolol is predominantly metabolized by CYP2D6, while paroxetine and fluoxetine are strong inhibitors of the enzyme.^11^, ^12^, ^13^ Consequently, co‐prescription of these drugs leads to the substantial increase of the blood concentration of metoprolol and potentially induces metoprolol‐related adverse drug reactions.^8^, ^10^, ^14^, ^15^

The frequent co‐administration of the drugs makes the clinical relevance of the DDI important to be determined, but so far real‐world data about its clinical consequences are sparse and conflicting. Some case reports indicated that the co‐medication of metoprolol and paroxetine/fluoxetine produces bradycardia and atrioventricular block in elderly.^10^, ^16^, ^17^ However, another observational study found that the risk of bradycardia in the older population with the interacting combination is not different from those without the combination.^7^

Therefore, the objective of this study was to investigate the clinical impact of such DDI by analyzing the effect of paroxetine or fluoxetine co‐prescription to the existing treatment with metoprolol on the metoprolol discontinuation rate or defined daily dose (DDD) among elderly. Earlier discontinuation and dose adjustment of metoprolol after the initiation of paroxetine/fluoxetine are used as indicators to represent the emergence of metoprolol related side effects.

**KEY POINTS**

- The combined use of metoprolol and paroxetine or fluoxetine can lead to CYP2D6‐mediated drug‐drug interaction and is frequently observed in older persons.
- Compared with the combination of metoprolol with citalopram, the metoprolol‐paroxetine/fluoxetine combination was not significantly associated with the risk of early discontinuation and dose adjustment of metoprolol.
- Compared with metoprolol‐mirtazapine, metoprolol‐paroxetine/fluoxetine combination was significantly associated with the risk of early discontinuation but not dose adjustment of metoprolol, notably among female older persons.

## 2 METHOD

### 2.1 Setting

This inception cohort study was performed using the University of Groningen prescription database IADB.nl which consists of over 1.2 million prescriptions since 1994 until 2015 from 60 community pharmacies in the Netherlands and covers approximately 600 000 anonymous individuals. The IADB provides information about the patients such as date of birth, gender and the prescribed drugs such as the date and the number of drugs being delivered to the patients, the Anatomical Therapeutic Chemical codes, the total number of DDD, duration of drug consumption, and the prescribers' code. The prescription data are updated every year, and the rate of prescription has been reported to represent the Dutch population generally.^18^ Prescription data from hospital and OTC drugs are not included in this database. The IADB.nl has been used as a reliable source of data for many pharmacoepidemiological researches.^19^, ^20^, ^21^

### 2.2 Study population

The study population were all elderly (≥60 years old) in the IADB who had ever been prescribed metoprolol (C07AB02) and had a first co‐prescription of paroxetine (N06AB05)/fluoxetine (N06AB03)/citalopram (N06AB04)/mirtazapine (N06AX11) during the period of January 1994 to September 2015. They had not been prescribed with the drugs and recorded in the IADB for at least 9 months before the first prescriptions. If the patients experienced several prescriptions of metoprolol, we included only the first time of prescription. All patients using antivirals for treatment of HCV infections (J05AP), interferon (L03AB), bile and liver therapy (A05), and drugs for alcohol dependence (N07BB) were excluded because they probably have hepatic problems, and these condition may influence the metabolic capacity of hepatic enzyme.^22^, ^23^, ^24^ Patients with any other antidepressant prescriptions (N06A) beside the studied drugs or patients with chronotropic drug prescriptions such as verapamil (C08DA01), diltiazem (C08DB01), and digoxin (C01AA05) or other CYP2D6 inhibitors in exposed and non‐exposed groups were excluded. Other CYP2D6 inhibitors comprised cimetidine (A02BA01), amiodarone (C01BD01), terbinafine (D01BA02), quinidine (C01BA01), bupropion (N06AX12), chlorpromazine (N05AA01), dexchlorpheniramine (R06AB02), clomipramine (N06AA04), doxorubicin (L01DB01), haloperidol (N05 AD01), levomepromazine (N05AA02), metoclopramide (A03FA01), mibefradil (C08CX01), moclobemide (N06AG02), ranitidine (A02BA02), ritonavir (J05AE03), sertraline (N06AB06), diphenhydramine (R06AA02), perphenazine (N05AB03), hydroxyzine (N05BB01), propafenone (C01BC03), mirabegron (G04BD12), cinacalcet (H05BX01), panobinostat (L01XX42), abiraterone (L02BX03), aripiprazole (N05AX12), doxepin (N06AA12), venlafaxine (N06AX16), duloxetine (N06AX21), methadone (N07 BC02), fluvoxamine (N06AB08), and tripelennamine (R06AC04).^25^

### 2.3 Exposed group and non‐exposed group

The exposure group was defined as metoprolol with a paroxetine/fluoxetine co‐prescription. The non‐exposed groups were defined as either metoprolol with citalopram or with mirtazapine co‐prescriptions. The date of the first metoprolol‐paroxetine/fluoxetine/citalopram/mirtazepine co‐prescription was defined as an index date. The combination can take place in 2 condition as follows: First, metoprolol and paroxetine/fluoxetine/citalopram/mirtazapine were co‐prescribed at the same start date. Second, paroxetine/fluoxetine/citalopram/mirtazapine were prescribed during the use of metoprolol.

Citalopram was chosen as a comparator because it is the most preferable drug of choice to be combined with metoprolol besides paroxetine/fluoxetine.^5^ However, because it is a weak inhibitor of CYP2D6 (Ki = 5.1 microM), we used mirtazapine (Ki = 41 microM) as a negative control because it has a very minimal CYP2D6 inhibitory activity and has no interaction with metoprolol.^9^, ^26^, ^27^, ^28^ As a comparison, paroxetine and fluoxetine, as potent inhibitors of CYP2D6, have Ki value = 0.15 and 0.60 microM, respectively.^26^ To see the impact of potential interaction of citalopram and metoprolol, we also compare the effect of the combination with the mirtazapine‐metoprolol combination (supplementary 2).

### 2.4 Outcomes

We assumed that the adverse drug reactions produced by the combination of metoprolol‐paroxetine/fluoxetine would make the prescribers to decide for either an early discontinuation or a dose adjustment of metoprolol. Therefore, we used these clinical outcomes as indicators of the adverse effect of the DDI. Early discontinuation was defined as stopped within 3 months and not re‐prescribed in a maximum period of 9 months after the index date. Dose adjustment was defined as having at least 50% DDD relative reduction of metoprolol between without and with paroxetine/fluoxetine/citalopram/mirtazapine. DDD of metoprolol with paroxetine/fluoxetine/citalopram/mirtazapine was obtained from the dose of metoprolol at the index date or during the combination or within 14 days after the stop date (the date in which the combination was discontinued). The latest was taken into account because the CYP2D6 inhibitory capacity of paroxetine/fluoxetine (norfluoxetine) may linger approximately 2 weeks after their discontinuation.^12^, ^29^, ^30^ This persistent inhibition may happen because paroxetine, fluoxetine, and norfluoxetine (main metabolite of fluoxetine, which also has a potent inhibitory effect on CYP2D6; Ki = 0.43 microM) can inhibit their own clearance; therefore, they have a long half‐life.^26^, ^31^, ^32^ DDD of metoprolol without paroxetine/fluoxetine/citalopram/mirtazapine was taken from the dose of metoprolol before the index date or the dose of metoprolol at least 2 weeks after the stop date.

### 2.5 Co‐variates

Potential confounders were age, sex, dose of metoprolol without paroxetine/fluoxetine/citalopram/mirtazapine, and the number of different types of prescribed medication 1 year before the index date. Complete list of Anatomical Therapeutic Chemicals that were checked can be found in the supplementary 1.

### 2.6 Statistical analysis

The Chi‐square test was used to compare the difference of gender distribution between exposed and non‐exposed groups. Independent Mann‐Whitney test was used to compare non‐normally distributed continuous variables (age, dose of metoprolol without paroxetine/fluoxetine/citalopram/ mirtazapine, and number of medications 1 year before the index date) of exposed and non‐exposed groups. The significant variable (*P* < 0.05) was included in the multivariate analysis to calculate the adjusted odds ratio (OR). Logistic regression analysis was applied to estimate adjusted risk estimates. An OR of more than one and the range of 95% of confidence interval (CI) not containing one indicated a statistically significant association between the co‐prescription of metoprolol‐paroxetine/fluoxetine to the outcomes. Statistical Program for Social Sciences version 24.0 for Windows was used to perform the statistical analysis.

## 3 RESULTS

**Figure 1:**
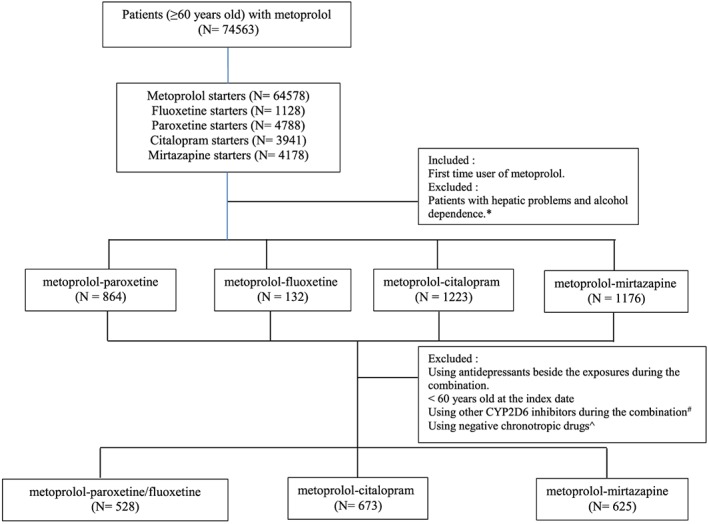
Flow diagram of the selection process for the study population. *Patients prescribed with antivirals for treatment of HCV infections (J05AP), interferon (L03AB), and bile and liver therapy (A05) were considered having hepatic problems. Patients prescribed with drugs used in alcohol dependence (N07BB) were considered as patients with alcohol dependence. #Other CYP2D6 inhibitors consist of cimetidine (A02BA01), amiodarone (C01BD01), terbinafine (D01BA02), quinidine (C01BA01), bupropion (N06AX12), chlorpromazine (N05AA01), dexchlorpheniramine (R06AB02), clomipramine (N06AA04), doxorubicin (L01DB01), haloperidol (N05AD01), levomepromazine (N05AA02), metoclopramide (A03FA01), mibefradil (C08CX01), moclobemide (N06AG02), ranitidine (A02BA02), ritonavir (J05AE03), sertraline (N06AB06), diphenhydramine (R06AA02), perphenazine (N05AB03), hydroxyzine (N05BB01), propafenone (C01BC03), mirabegron (G04BD12), cinacalcet (H05BX01), panobinostat (L01XX42), abiraterone (L02BX03), aripiprazole (N05AX12), doxepin (N06AA12), venlafaxine (N06AX16), duloxetine (N06AX21), methadone (N07BC02), fluvoxamine (N06AB08), and tripelennamine (R06AC04). ^Patients using chronotropic drugs such as verapamil (C08DA01), diltiazem (C08DB01), and digoxin (C01AA05) [Colour figure can be viewed at http://wileyonlinelibrary.com]

The number of patients included as metoprolol‐paroxetine/fluoxetine group, metoprolol‐citalopram group, and metoprolol‐mirtazapine group were 528, 673, and 625, respectively (Figure 1). The large majority were female in each group (more than 60%). The median of age was significantly different between the exposed (71.37 years [IQR = 13]) and non‐exposed groups (76.38 [IQR = 14.40] and 76.15 [IQR = 12.75] for metoprolol‐citalopram and metoprolol‐mirtazapine group, respectively). Meanwhile, DDD of metoprolol at baseline was comparable among groups (approximately 0.5 DDD). Lastly, the number of different types of prescribed medication 1 year before the index date was significantly lower in exposed (7.00 [IQR = 4.00]) than non‐exposed groups (7.00 [IQR = 4.00] and 8.00 [IQR = 5.00] for metoprolol‐citalopram and metoprolol‐mirtazapine, consecutively) (Table 1).

**Table 1:** Baseline characteristics of metoprolol‐paroxetine/fluoxetine, metoprolol‐citalopram, and metoprolol‐mirtazapine

Variable	Metoprolol‐Paroxetine/Fluoxetine (*N* = 528)	Metoprolol‐Citalopram (*N* = 673)	*P*‐Value	Metoprolol‐ Mirtazapine (*N* = 625)	*P*‐Value
Age in year, median (IQR)	71.37 (13)	76.38 (14.40)	*P* < 0.01	76.15 (12.75)	*P* < 0.01
Gender, *N* woman (%)	356 (67.40)	447 (66.40)	*P* = 0.68	420 (67.20)	*P* = 0.89
Number of medications 1 year before index date, median (IQR)	7.00 (4.00)	7.00 (4.00)	*P* < 0.01	8.00 (5.00)	*P* < 0.01
Dose of metoprolol without exposures in DDD, median (IQR)*	0.56 (0.33)	0.52 (0.33)	*P* = 0.47	0.49 (0.33)	*P* = 0.33
*DDD at age ≤ 70*	0.61 (0.34)	0.51 (0.33)	*P* = 0.07	0.52 (0.34)	*P* = 0.16
*DDD at age 71–80*	0.57 (0.36)	0.55 (0.34)	*P* = 0.94	0.51 (0.33)	*P* = 0.46
*DDD at age ≥ 81*	0.36 (0.33)	0.49 (0.33)	*P* < 0.05	0.47 (0.33)	*P* = 0.06

The risk of the discontinuation and dose adjustment of metoprolol was not significantly different between metoprolol‐paroxetine/fluoxetine and metoprolol‐citalopram (adjusted OR 1.07 [95% CI 0.77‐1.48] and adjusted OR 0.87 [95% CI 0.57‐1.33], respectively). The subgroup analysis by age and gender presented comparable results (Table 2).

**Table 2:** Outcomes for metoprolol‐paroxetine/fluoxetine and metoprolol‐citalopram

Outcomes	Metoprolol‐Paroxetine/Fluoxetine		Metoprolol‐Citalopram		Crude OR (95%CI)	Adjusted OR^a^ (95%CI)
	*n*	%	*n*	%		
*Overall*	*N* = 528		*N* = 673			
Discontinuation	80	15.20	109	16.20	0.92 (0.67–1.26)	1.07 (0.77–1.48)
Dose adjustment	42	8.00	63	9.40	0.84 (0.56–1.26)	0.87 (0.57–1.33)
*Age group*						
*≤70*	*N* = 243		*N* = 192			
Discontinuation	32	13.20	28	14.60	0.89 (0.51–1.54)	0.86 (0.49–1.49)
Dose adjustment	20	8.20	16	8.30	0.99 (0.49–1.96)	0.99 (0.49–1.97)
*71–80*	*N* = 197		*N* = 243			
Discontinuation	28	14.20	26	10.70	1.38 (0.78–2.45)	1.35 (0.76–2.39)
Dose adjustment	16	8.10	23	9.50	0.85 (0.43–1.65)	0.87 (0.44–1.70)
*≥81*	*N* = 88		*N* = 238			
Discontinuation	20	22.70	55	23.10	0.98 (0.55–1.75)	1.06 (0.58–1.92)
Dose adjustment	6	6.80	24	10.10	0.65 (0.26–1.65)	0.66 (0.26–1.70)
*Gender*						
*Men*	*N* = 171		*N* = 226			
Discontinuation	29	17.00	43	19.00	0.87 (0.52–1.46)	0.98 (0.57–1.67)
Dose adjustment	12	7.00	26	11.50	0.58 (0.28–1.19)	0.62 (0.30–1.30)
*Women*	*N* = 356		*N* = 448			
Discontinuation	50	14.00	66	14.80	0.94 (0.63–1.40)	1.15 (0.76–1.74)
Dose adjustment	30	8.40	37	8.30	1.02 (0.62–1.69)	1.09 (0.65–1.84)

a Adjusted for age and number of medications 1 year before index date.

Compared with the metoprolol‐mirtazapine group, the metoprolol‐paroxetine/fluoxetine group had approximately 43% significantly higher risk to experience the early discontinuation (adjusted OR = 1.43, 95% CI [1.01‐2.02]) but not to the dose adjustment of metoprolol (adjusted OR = 1.00, 95% CI [0.65‐1.54]) (Table 3). After stratification on age, no clear difference was found between patients. However, subgroup analysis by gender indicated that women, but not men, using metoprolol‐paroxetine/fluoxetine were significantly at risk having the early discontinuation of metoprolol compared with the non‐exposure group (women: adjusted OR 1.62 [95% CI 1.03‐2.53], men: adjusted OR 1.23 [95% CI 0.70‐2.17]). Yet, they had a comparable result in the risk of dose adjustment.

**Table 3:** Outcomes of metoprolol‐paroxetine/fluoxetine and metoprolol‐mirtazapine

Outcomes	Metoprolol‐Paroxetine/Fluoxetine		Metoprolol‐ Mirtazapine		Crude OR (95%CI)	Adjusted OR^a^ (95%CI)
	n	%	n	%		
*Overall*	N = 528		N = 625			
Discontinuation	80	15.20	79	12.60	1.23 (0.88–1.72)	1.43 (1.01–2.02)*
Dose adjustment	42	8.00	54	8.60	0.91 (0.60–1.39)	1.00 (0.65–1.54)
*Age group*						
*≤ 70*	N = 243		*N* = 193			
Discontinuation	32	13.20	18	9.30	1.47 (0.80–2.72)	1.57 (0.85–2.92)
Dose adjustment	20	8.20	12	6.20	1.35 (0.64–2.84)	1.36 (0.65–2.87)
*71–80*	N = 197		*N* = 241			
Discontinuation	28	14.20	30	12.40	1.16 (0.67–2.03)	1.22 (0.69–2.13)
Dose adjustment	16	8.10	23	9.50	0.84 (0.43–1.63)	0.89 (0.45–1.76)
*≥ 81*	*N* = 88		*N* = 191			
Discontinuation	20	22.70	31	16.20	1.52 (0.81–2.85)	1.61 (0.85–3.05)
Dose adjustment	6	6.80	19	9.90	0.66 (0.25–1.72)	0.74 (0.28–1.94)
*Gender*						
*Men*	N = 171		*N* = 205			
Discontinuation	29	17.00	34	16.60	1.03 (0.59–1.77)	1.23 (0.70–2.17)
Dose adjustment	12	7.00	17	8.30	0.84 (0.39–1.80)	1.02 (0.61–1.72)
*Women*	*N* = 356		*N* = 420			
Discontinuation	50	14.00	45	10.70	1.36 (0.88–2.09)	1.62 (1.03–2.53)*
Dose adjustment	30	8.40	37	8.80	0.95 (0.58–1.58)	1.02 (0.46–2.26)

a Adjusted for age and number of medications 1 year before index date.

* *P* < 0.05.

The results of citalopram‐metoprolol and mirtazapine‐metoprolol comparison showed that citalopram‐metoprolol is associated with 34% higher risk of early discontinuation of metoprolol (adjusted OR = 1.34, 95% CI [0.98‐1.83]) and especially for women, it has a 44% relative increase in the risk of early discontinuation of metoprolol (adjusted OR = 1.44, 95% CI [0.96‐2.16]) (*P* value = 0.07) (supplementary 2).

## 4 DISCUSSION

Our study is the first cohort study to provide evidence of the effect of the metoprolol‐paroxetine/fluoxetine co‐prescription in elderly using community pharmacy prescription data. We found that the risk of discontinuation and dose adjustment of metoprolol in the metoprolol‐paroxetine/fluoxetine combination is not significantly different from the metoprolol‐citalopram combination but had a 43% higher risk of early discontinuation of metoprolol compared with the metoprolol‐mirtazapine group.

The result of the metoprolol‐paroxetine/fluoxetine and metoprolol‐citalopram comparison is in line with a case control study performed by Kurdyak PA et al.^7^ They reported that compared with the combination of non‐inhibiting CYP2D6 antidepressants‐metoprolol, there was no significant association of metoprolol‐paroxetine/fluoxetine with the risk of bradycardia in elderly. Yet, this study has some limitations. The first limitation is that they did not consider the weak CYP2D6 inhibitory capacity of citalopram as well as fluvoxamine in their analysis.^9^, ^11^, ^26^, ^33^, ^34^ Although citalopram is considered to be safely combined with metoprolol, it is still able to increase the AUC of metoprolol approximately 2 to 3 times.^9^, ^33^, ^35^ This weak inhibition may be important in the older people because of the age‐related physiological changes.

Although the metabolic function of CYP2D6 is reported not to decline by aging, other CYPs such as CYP1A2, CYP2C9, CYP2C19, and CYP3A4 do.^36^, ^37^, ^38^ This is important in 2 aspects. Firstly, metoprolol is mainly metabolized by CYP2D6 and secondarily metabolized by CYP3A4. The reduced function of CYP3A4 in the elderly leads to a more important role of CYP2D6 in metabolizing metoprolol as a form of compensatory mechanism.^39^ Therefore, the weak inhibition of CYP2D6 may increase the blood concentration of metoprolol further in the elderly population. Secondly, the concentration of citalopram, metabolized mainly by CYP2C19, may be relatively higher in the older population thereby increasing the inhibition of CYP2D6. It is estimated that there is an increase of approximately 130% of the citalopram plasma concentration in elderly compared with the younger population.^33^

The second limitation, which also may explain our results, is that citalopram itself is associated with bradycardia which is reported more common in the older (>65 years) than in the younger population.^40^, ^41^, ^42^, ^43^, ^44^ This side effect may also be more apparent in the elderly using metoprolol. Hence, the result of citalopram‐metoprolol co‐prescription depends not only on the mild CYP2D6 inhibitory effect of citalopram but also on the side effects of citalopram.

To gain insight into the potential bias induced by those limitations, we used a combination of metoprolol‐mirtazapine as a negative control for metoprolol‐paroxetine/fluoxetine. Metoprolol and mirtazapine is reported to have no interaction; therefore, it may provide a good contrast for the interaction effect of metoprolol‐paroxetine/fluoxetine.^9^, ^28^ As expected, the results indicated that metoprolol‐paroxetine/fluoxetine co‐prescriptions had a significant risk of having early discontinuation of metoprolol.

We also found that the exposed group was a little younger than controls in the baseline characteristics. If anything such a difference may work against finding differences, we adjusted for differences to have the final adjusted odd ratio (OR).

Subgroup analysis by gender indicated that women using the interacting combination have a significant 62% increased risk of experiencing early discontinuation of metoprolol compared with those using the non‐interacting combination. Meanwhile, there was no significant difference in the risk of having the outcome in the male population. One possible explanation is the difference in the body mass index (BMI) between men and women. In this study, we did not have the information about the BMI of patients and whether the prescribed doses of metoprolol were normalized to the BMI. Therefore, it is possible that the unadjusted dose of metoprolol may be the culprit. Our results are in line with the study reported by Sharma et al on the interaction between metoprolol and diphenhydramine.^45^ They found that diphenhydramine increases the AUC value of metoprolol significantly higher in women than men, but the differences still remain even after the dose correction by body weight. Another possibility is the differences in the baseline activity of CYP2D6 between males and females. However, the studies about the differences are conflicting. Walle et al and Kashuba et al reported that gender has no influence on the metabolic activity of CYP2D6.^46^, ^47^ Meanwhile, other studies reported that women have a faster CYP2D6 metabolic activity compared with men.^48^, ^49^ Borobia et al also reported that the differences are existing, yet are not clinically relevant.^50^ More studies are needed to investigate the underlying factors causing the differences in the effect of interaction.

Some limitations are worth to be mentioned in this study. First, there was no real information whether the patients were taking metoprolol as prescribed. Second, we had no data related to heart rate, blood pressures, or bradycardia as the best indicators to assess the side effects of metoprolol. Third, we did not check the metoprolol plasma concentration which can properly indicate the impact of interaction. Fourth, there was no information about the patient specific genetic status of CYP2D6. This is important because individuals with different CYP2D6 genotypes may have a different response toward the interaction.^39^ Goryachkina et al reported that among 17 patients with acute myocardial infarction treated with the combination of metoprolol‐paroxetine, there were 2 patients experiencing dose adjustments due to hypotension and bradycardia. Interestingly, these 2 patients were intermediate metabolizer for CYP2D6.^6^ The reduced metabolic activity of CYP2D6 might increase the exposure of metoprolol, and this condition was corroborated by the strong inhibition of CYP2D6 by paroxetine which results in unexpected higher metoprolol plasma concentration. Furthermore, patients with ultra‐rapid metabolizer (UM) genotype of CYP2D6 may also theoretically have a high risk in experiencing metoprolol‐related adverse reactions. The CYP2D6 UM patients have a greater metabolic rate of metoprolol than CYP2D6 normal metabolizers. Hence, it has been suggested to increase the dose of metoprolol 2.5 times the normal daily dose for these patients.^51^ It has been reported that the plasma concentration of paroxetine in CYP2D6 UM patients is very low or undetectable; therefore, the interaction is unlikely to exist, but a different scenario takes place for fluoxetine.^52^ It is also extensively metabolized by CYP2D6 to its metabolite, norfluoxetine, but this metabolite also has a potent CYP2D6 inhibitory capacity.^13^, ^26^, ^53^ Consequently, norfluoxetine may impair the degradation of metoprolol and increase the AUC value of metoprolol in these patients. The combination of metoprolol‐fluoxetine in CYP2D6 UM individuals may have a high risk of developing metoprolol‐related side effects. Therefore, it might be interesting to further investigate the outcomes of the interacting drugs in different genotype statuses. Fifth, besides the effect of interaction, there are other factors that may contribute to metoprolol discontinuation. Girouard et al reported that elderly patients who get β‐blocker prescription after the first heart failure diagnosis have a tendency to discontinue their treatment (median duration from the start of β‐blocker prescription until the discontinuation is approximately 6 months) if they have COPD, asthma, dementia, and more than 9 physician visits with the reported increased risk approximately 8%, 9%, 13%, and 14%, respectively.^54^ We do not have information about the number of medical visits in the IADB database. However, for the comorbidities, we tried to control them by comparing the distribution of the diseases in the exposed and non‐exposed groups and then, adjust the differences in the multivariate analysis (supplementary data 3). Asthma or COPD was defined as patients having a prescription for drugs used to treat obstructive airway diseases (R03). Dementia was defined as patients being prescribed with anti‐dementia drugs (N06D). We found that dementia was more prevalent in the exposed groups and COPD/asthma was not statistically different. After the adjustment of the differences in the variable distributions, we observed that the adjusted OR was comparable with the main results in both comparisons of metoprolol‐paroxetine/fluoxetine and metoprolol‐citalopram, and metoprolol‐paroxetine/fluoxetine and metoprolol‐mirtazapine for the 2 outcomes (supplementary data 3). Therefore, we concluded that dementia and COPD/asthma have no substantial influence on the outcomes.

In the Netherlands, despite the fact that computerized DDI alerting systems have been incorporated in the electronic prescription systems and applied before the dispensing process in the pharmacy, the combination of metoprolol‐paroxetine/fluoxetine is still common in older patients.^5^, ^55^, ^56^, ^57^ One possible reason is that there is a conflicting response of the applied surveillance systems in assessing the DDI because of the contrasting evidence in the clinical consequences of metoprolol and paroxetine/fluoxetine interaction.^5^ The G‐standard, a product from the “Royal Dutch Association for the Advancement of Pharmacy” (KNMP) and used by approximately 45% of the pharmacies, does alert the interaction, but the Pharmabase, a product from the Health Base Foundation and used by approximately 55% of the pharmacies, has been stopping alerting the combination since 2005.^5^ This case should be solved because if the DDI is clinically relevant, the decision of not alerting the DDI may harm the population. However, if the DDI is not clinically relevant, alerting the DDI may lead to the “alert fatigue” problem as the important drawback of DDI surveillance systems. The sensitivity and specificity of the DDI alerting systems are the main issues in the application of such surveillance system.^58^, ^59^, ^60^ Therefore, this study is important because it can add evidence regarding the effect of the DDI so that it may increase the accuracy of the DDI alerting systems.^60^, ^61^

In this study, we also compared the citalopram‐metoprolol combination and the negative control. It seems that metoprolol which was co‐prescribed with citalopram was likely to be discontinued earlier than metoprolol combined with mirtazapine especially in the females group (supplementary 2). More research is required to elucidate the potential impact of the combination on metoprolol treatment.

As a conclusion, the initiation of paroxetine/fluoxetine in metoprolol users in elderly, especially among female patients, was associated with the risk of experiencing early discontinuation of metoprolol. Hence, we recommend avoiding this combination in clinical practice because a more effective and safety drug combination is available.

## ETHICS STATEMENT

The authors state that no ethical approval was needed.

## CONFLICT OF INTEREST

None declared.

## Supporting information

Supporting Information
