# Supplementary material for: Discontinuation and dose adjustment of metoprolol after metoprolol‐paroxetine/fluoxetine co‐prescription in Dutch elderly
Source: Pharmacoepidemiol Drug Saf. 2018 Mar 24;27(6):621–9. doi: 10.1002/pds.4422 (PMC6001522; doi:10.1002/pds.4422)
Supplement: Supplementary file 1 — Supporting Information [file PDS-27-621-s001.docx]

**Discontinuation and Dose Adjustments of Metoprolol after Metoprolol- Paroxetine/Fluoxetine Co-prescriptions in Dutch Elderly**

Muh. Akbar Bahar^1,2^, Yuanyuan Wang^1^, Jens H. Bos^1^, Bob Wilffert^1,3^, Eelko Hak^1^

*^1^Groningen Research Institute of Pharmacy, Department of PharmacoTherapy, -Epidemiology & -Economics, University of Groningen, Groningen, the Netherlands*

*^2^Faculty of Pharmacy, Hasanuddin University, Makassar, Indonesia*

*^3^Dept. of Clinical Pharmacy and Pharmacology University of Groningen, University Medical Center Groningen, Groningen, the Netherlands*

†Corresponding author: Muh. Akbar Bahar, Department of PharmacoTherapy, -Epidemiology & -Economics, University of Groningen, Groningen, the Netherlands. Tel: +31 50 3632954. Email address: m.a.bahar@rug.nl.

ORCHID ID of corresponding author: orcid.org/0000-0002-6582-5615

Supplementary 1

List of drugs to be checked one year before the index date:

C01 (cardiac therapy), C02 (antihypertensives), C03 (diuretics), C04 (peripheral vasodilators), C05 (vasoprotectives), C08 (calcium channel blockers), C09 (agents acting on the renin-angiotensin system), C10 (lipid modifying agents), A01 (stomatological preparations), A02 (drugs for acid related disorders), A03 (drugs for functional gastrointestinal disorders), A04 (antiemetics and antinauseants), A05 (bile and liver therapy), A06 (drugs for constipation), A07 (antidiarrheals, intestinal antiinflammatory/antiinfective agents), A08 (antiobesity preparations, excl. diet products), A09 (digestives, incl. enzymes), A10 (drugs used in diabetes), A11 (vitamins), B01 (antithrombotic agents), B02 (antihemorrhagics), B03 (antianemic preparations), B05 (blood substitutes and perfusion solutions), B06 (other hematological agents), N02 (analgesics), N03 (antiepileptics), N04 (anti-parkinson drugs), N05 (psycholeptics), N07 (other nervous system drugs), J01 (antibacterials for systemic use), J02 (antimycotics for systemic use), J04 (antimycobacterials), J05 (antivirals for systemic use), J06 (immune sera and immunoglobulins), J07 (vaccines), M01 (antiinflammatory and antirheumatic products), M03 (muscle relaxants), M04 (antigout preparations), M05 (drugs for treatment of bone diseases), M09 (other drugs for disorders of the musculo-skeletal system), P01 (antiprotozoals), P02 (anthelmintics), D01B (antifungals for systemic use), R02 (throat preparations), R03 (drugs for obstructive airway diseases), R05 (cough and cold preparations), R06 (antihistamines for systemic use), R07 (other respiratory system products), L01 (antineoplastic agents), L02 (endocrine therapy), L03 (immunostimulants), L04 (immunosuppressants), and V03A (antidotes, iron chelating agents, drugs for treatment of hyperkalemia and hyperphosphatemia, detoxifying agents for antineoplastic treatment, drugs for treatment of hypercalcemia, drugs for treatment of hypoglycemia, tissue adhesives, drugs for embolization, medical gases, and nerve depressants).

**Supplementary 2**

**Table 1.** Baseline characteristics of Metoprolol-Citalopram and Metoprolol-Mirtazapine.

| **Variable** | **Metoprolol-Citalopram (N = 673)** | **Metoprolol-**  **Mirtazapine (N = 625)** | **P-value** |
| --- | --- | --- | --- |
| Age in year, median (IQR) | 76.38 (14.40) | 76.15 (12.75) | P = 0.21 |
| Gender, N woman (%) | 447 (66.40) | 420 (67.20) | P = 0.76 |
| Number of medications 1 year before index date, median (IQR) | 7.00 (4.00) | 8.00 (5.00) | P = 0.36 |
| Dose of metoprolol without exposures in DDD, median (IQR)* | 0.52 (0.33) | 0.49 (0.33) | P = 0.72 |
| *DDD at age ≤ 70* | 0.51 (0.33) | 0.52 (0.34) | P = 0.74 |
| *DDD at age 71 - 80* | 0.55 (0.34) | 0.51 (0.33) | P = 0.48 |
| *DDD at age ≥ 81* | 0.49 (0.33) | 0.47 (0.33) | P = 0.76 |

**Table 2.** Outcomes of Metoprolol-Citalopram and Metoprolol-Mirtazapine

| Outcomes | Metoprolol-Citalopram | | Metoprolol- Mirtazapine | | | OR (95%CI) |
| --- | --- | --- | --- | --- | --- | --- |
|  | n | % | n | | % |  |
| *Overall* | N= 673 | | N= 625 | | |  |
| Discontinuation | 109 | 16.20 | 79 | | 12.60 | 1.34 (0.98-1.83)* |
| Dose adjustment | 63 | 9.40 | 54 | | 8.60 | 1.09 (0.75-1.59) |
| *Age group* |  |  |  | |  |  |
| *≤ 70* | N = 192 | | N = 193 | | |  |
| Discontinuation | 28 | 14.60 | 18 | | 9.30 | 1.66 (0.85-2.92) |
| Dose adjustment | 16 | 8.30 | 12 | | 6.20 | 1.37 (0.63-2.98) |
| *71 - 80* | N = 243 | | N = 241 | | |  |
| Discontinuation | 26 | 10.70 | 30 | | 12.40 | 0.84 (0.48-1.47) |
| Dose adjustment | 23 | 9.50 | 23 | | 9.50 | 0.99 (0.54-1.82) |
| *≥ 81* | N = 238 | | N = 191 | | |  |
| Discontinuation | 55 | 23.10 | 31 | | 16.20 | 1.55 (0.95-2.53) |
| Dose adjustment | 24 | 10.10 | 19 | | 9.90 | 1.02 (0.54-1.92) |
| *Gender* |  |  |  | |  |  |
| *Men* | N = 226 | | N= 205 | | |  |
| Discontinuation | 43 | 19.00 | 34 | 16.60 | | 1.18 (0.72-1.94) |
| Dose adjustment | 26 | 11.50 | 17 | 8.30 | | 1.44 (0.76-2.73) |
| *Women* | N = 448 | | N = 420 | | |  |
| Discontinuation | 66 | 14.80 | 45 | 10.70 | | 1.44 (0.96-2.16) * |
| Dose adjustment | 37 | 8.30 | 37 | 8.80 | | 0.93 (0.58-1.50) |

*P=0.07

**Supplementary 3**

**Table 1.** Comparison of Potential Comorbidities in the Metoprolol-Paroxetine/Fluoxetine, Metoprolol-Citalopram, and Metoprolol-Mirtazapine groups.

| **Variable** | **Metoprolol-Paroxetine/Fluoxetine (N = 528)** | **Metoprolol-Citalopram (N = 673)** | **P-value** | **Metoprolol-**  **Mirtazepine (N = 625)** | **P-value** |
| --- | --- | --- | --- | --- | --- |
| Cancer, N yes (%) | 8.00 (1.50) | 16.00 (2.40) | P = 0.29 | 17.00 (2.70) | P = 0.16 |
| Asthma or COPD, N yes (%) | 72.00 (13.60) | 102 (15.20) | P = 0.46 | 94 (15.00) | P = 0.49 |
| Dementia, N yes (%) | 3.00 (0.60) | 21.00 (3.10) | P < 0.05 | 13.00 (2.10) | P < 0.05 |

Cancer was defined as patients having antineoplastic agents (L01) prescriptions; Asthma or COPD was defined as patients having prescription for drugs used to treat obstructive airway diseases (R03); Dementia was defined as patients being prescribed with anti-dementia drugs (N06D).

**Table 2.** Outcomes for Metoprolol-Paroxetine/Fluoxetine and Metoprolol-Citalopram After Being Adjusted by Age, Number of Medications 1 year Before Index Date and Dementia

| **Outcomes** | **Metoprolol-Paroxetine/Fluoxetine** | | **Metoprolol-Citalopram** | | **Adjusted OR**^#^ **(95%CI)** |  |
| --- | --- | --- | --- | --- | --- | --- |
|  | n | % | n | % |  |  |
| *Overall* | N= 528 | | N= 673 | |  |  |
| Discontinuation | 80 | 15.20 | 109 | 16.20 | 1.05 (0.758-1.455) |  |
| Dose adjustment | 42 | 8.00 | 63 | 9.40 | 0.86 (0.567-1.314) |  |
| *Age group* |  |  |  |  |  |  |
| *≤70* | N = 243 | | N = 192 | |  |  |
| Discontinuation | 32 | 13.20 | 28 | 14.60 | 0.88 (0.507-1.540) |  |
| Dose adjustment | 20 | 8.20 | 16 | 8.30 | 0.98 (0.491-1.954) |  |
| *71 - 80* | N = 197 | | N = 243 | |  |  |
| Discontinuation | 28 | 14.20 | 26 | 10.70 | 1.30 (0.732-2.314) | |
| Dose adjustment | 16 | 8.10 | 23 | 9.50 | 0.87 (0.441-1.702) | |
| *≥81* | N = 88 | | N = 238 | |  |  |
| Discontinuation | 20 | 22.70 | 55 | 23.10 | 1.03 (0.568-1.878) | |
| Dose adjustment | 6 | 6.80 | 24 | 10.10 | 0.64 (0.247-1.638) | |
| *Gender* |  |  |  |  |  | |
| *Men* | N = 171 | | N= 226 | |  | |
| Discontinuation | 29 | 17.00 | 43 | 19.00 | 0.96 (0.562-1.645) | |
| Dose adjustment | 12 | 7.00 | 26 | 11.50 | 0.61 (0.294-1.275) | |
| *Women* | N = 356 | | N = 448 | |  | |
| Discontinuation | 50 | 14.00 | 66 | 14.80 | 1.13 (0.746-1.722) | |
| Dose adjustment | 30 | 8.40 | 37 | 8.30 | 1.09 (0.645-1.844) | |

^#^ Adjusted for age, number of medications 1 year before index date and dementia

**Table 3.** Outcomes of Metoprolol-Paroxetine/Fluoxetine and Metoprolol-Mirtazepine After Being Adjusted by Age, Number of Medications 1 year Before Index Date and Dementia

| Outcomes | Metoprolol-Paroxetine/Fluoxetine | | Metoprolol- Mirtazepine | | Adjusted OR^#^ (95%CI) |
| --- | --- | --- | --- | --- | --- |
|  | n | % | n | % |  |
| *Overall* | N= 528 | | N= 625 | |  |
| Discontinuation | 80 | 15.20 | 79 | 12.60 | 1.42 (1.003-2.009)* |
| Dose adjustment | 42 | 8.00 | 54 | 8.60 | 1.00 (0.649-1.542) |
| *Age group* |  |  |  |  |  |
| *≤ 70* | N = 243 | | N = 193 | |  |
| Discontinuation | 32 | 13.20 | 18 | 9.30 | 1.56 (0.839-2.887) |
| Dose adjustment | 20 | 8.20 | 12 | 6.20 | 1.35 (0.641-2.845) |
| *71 - 80* | N = 197 | | N = 241 | |  |
| Discontinuation | 28 | 14.20 | 30 | 12.40 | 1.19 (0.683-2.100) |
| Dose adjustment | 16 | 8.10 | 23 | 9.50 | 0.89 (0.454-1.765) |
| *≥ 81* | N = 88 | | N = 191 | |  |
| Discontinuation | 20 | 22.70 | 31 | 16.20 | 1.62 (0.856-3.074) |
| Dose adjustment | 6 | 6.80 | 19 | 9.90 | 0.73 (0.276-1.923) |
| *Gender* |  |  |  |  |  |
| *Men* | N = 171 | | N= 205 | |  |
| Discontinuation | 29 | 17.00 | 34 | 16.60 | 1.23 (0.700-2.169) |
| Dose adjustment | 12 | 7.00 | 17 | 8.30 | 1.03 (0.610-1.736) |
| *Women* | N = 356 | | N = 420 | |  |
| Discontinuation | 50 | 14.00 | 45 | 10.70 | 1.58 (1.008-2.473)* |
| Dose adjustment | 30 | 8.40 | 37 | 8.80 | 1.03 (0.610-1.736) |

^#^Adjusted for age, number of medications 1 year before index date and dementia; *P<0.05
